# Expanding Access to Orthopedic Trauma Care: Evaluation of a Task‐Sharing Model With a Remote Quality Assessment Tool for Open Tibia Shaft Fractures in Malawi

**DOI:** 10.1002/wjs.70234

**Published:** 2026-02-03

**Authors:** Leonard Banza, Olaf Bach, Faith Moyo, Claude Martin, William Harrison

**Affiliations:** ^1^ Department of Trauma and Orthopaedic Surgery Lilongwe Institute of Orthopaedics and Neurosurgery (LION) Lilongwe Malawi; ^2^ Department of Trauma and Orthopaedic Surgery Zomba Central Hospital Zomba Malawi; ^3^ Department of International Public Health Liverpool School of Tropical Medicine Liverpool UK; ^4^ AO Alliance Foundation Chur Switzerland; ^5^ Countess of Chester Hospital NHS Foundation Trust Chester UK

**Keywords:** external fixation, LMICs, Malawi, open tibia fracture, quality assessment, task‐sharing

## Abstract

**Background:**

Timely care for open tibia fractures remains difficult in low‐resource settings. We evaluated a task‐sharing model in Malawi in which trained orthopedic clinical officers (OCOs) delivered external fixation supported by a remote quality assessment tool.

**Methods:**

We conducted a prospective implementation evaluation across one central and four district hospitals (May 2023–July 2024). The intervention bundled OR refurbishment assured external fixator supply, refresher training, mentoring (on‐site and remote), national guideline reinforcement, and a novel Fracture Fixation Assessment Tool for External Fixation (FFATEF). OCOs submitted postoperative radiographs and construct photographs for scoring across four domains (reduction, stability, implantation, and surgical impression; total 0–12 and satisfactory ≥ 8). Nonparametric tests compared performance by the hospital type; temporal trends were assessed with Spearman correlation.

**Results:**

Forty‐seven patients (89% male and mean age 32.3 years) were treated (central: *n* = 28 and district: *n* = 19). The central hospital managed more severe injuries (Gustilo IIIA/B 69.6% vs. 15.8%). Median FFATEF scores were higher at the central hospital (10.0 [IQR 9–11]) than district hospitals (6.0 [5, 6, 7, 8], *p* < 0.001). Satisfactory scores (≥ 8) occurred in 93% of central versus 32% of district cases. Central scores improved over time (*ρ* = 0.52; *p* = 0.005) whereas district scores were unchanged (*ρ* = 0.15; *p* = 0.540). Preoperative antibiotic compliance was 100% at the central versus 47% at district hospitals.

**Conclusions:**

When embedded within integrated surgical teams and adequate infrastructure, task sharing for open fracture external fixation yielded satisfactory technical performance but lagged district‐level implementation despite training. Comprehensive institutional support—mentoring intensity, equipment, supply chains, and referral adherence—is likely required for safe scale‐up. Validation of FFATEF against clinical outcomes and economic evaluation of delivery models are priorities.

## Graphical abstract

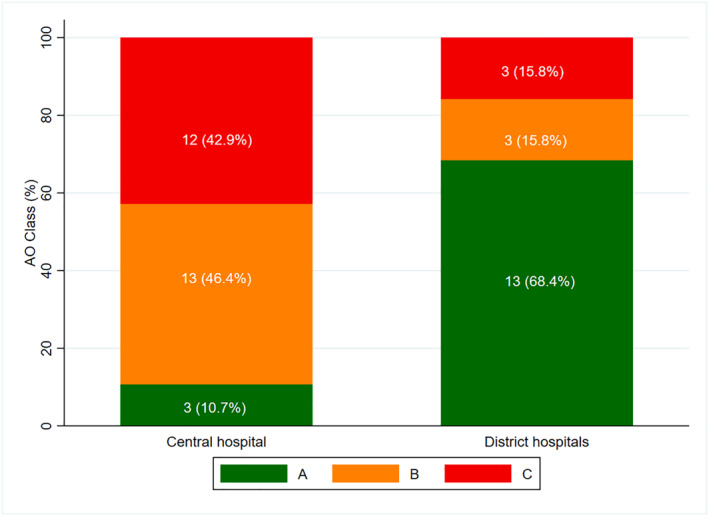

## 1 Introduction

Open fractures are bellwether conditions for emergency surgical capacity and are highly time‐sensitive [1, 2]. Access to surgical care in low‐ and middle‐income countries is constrained by multiple contextual challenges [3], and open tibia fractures represent a significant burden in settings such as Malawi [4]. Task sharing—collaborative care by mixed cadres under supervision—has been proposed to expand access in low‐ and middle‐income countries [5, 6, 7, 8, 9]. In Malawi, orthopedic clinical officers (OCOs) have delivered essential orthopedic care for over two decades [10], yet evidence on their performance for complex trauma outside tertiary centers remains limited [11]. We report a prospective evaluation of a bundled task‐sharing program for open tibia shaft fractures across five hospitals, incorporating a remote quality assessment tool to guide mentoring.

## 2 Methods

Study design and setting: During prospective implementation evaluation in one central (Zomba) and four district hospitals (Dowa, Machinga, Ntcheu, and Salima) from May 2023 to July 2024, it was found that eligible cases were skeletally mature patients with open tibia fractures.

Intervention: Operating room refurbishment, assured external fixator supply chain [12], refresher training for OCOs [13], reinforcement of national guidelines [14, 15], a multimodal mentoring package (quarterly visits, remote reviews within 72 h, case conferences, and on‐call phone support), and the FFATEF remote assessment tool were involved.

FFATEF: Adapted from the Fracture Fixation Assessment Tool [16, 17], FFATEF scores four domains—reduction, stability, implantation, and overall surgical impression—each 0–3; total 0–12 with satisfactory performance defined a priori as ≥ 8 (Table 1). OCOs uploaded postoperative AP/lateral radiographs and construct photos plus AO/OTA [18] and Gustilo [19] classifications and intent (temporary vs. definitive). A national trauma surgeon performed all primary ratings; a second surgeon independently rated a subset (*κ* = 0.76) indicating substantial agreement.

**TABLE 1:** Fracture fixation assessment tool for external fixation (FFATEF): domains and scoring anchors (0–3).

Domain	0 (Poor)	1 (Fair)	2 (Good)	3 (Excellent)
Reduction	Gross malalignment (> 10°/obvious translation)	Residual deformity 6°–10° or translation	≤ 5° angulation and minimal translation	Anatomic alignment and axes restored
Stability	Insecure frame and obvious looseness	Some instability and questionable pin spread	Adequate stability and acceptable pin spread	Rigid construct and optimal pin spread and triangulation
Implantation	Pins near wound and multiple avoidable conflicts	Suboptimal pin placement and minor conflicts	Good pin position and safe corridors mostly respected	Ideal pin position, safe corridors, and soft tissues protected
Surgical impression	Poor technique and contamination risk	Technique acceptable with notable issues	Competent technique and minor issues only	Flawless technique and meticulous execution

Outcomes and analysis: Primary outcome was the total FFATEF score. Nonparametric statistics (Mann–Whitney U and Fisher exact) compared sites; Spearman *ρ* tested trends. An ordinal logistic model explored correlates of satisfactory performance controlling for injury severity and time. Analyses used Stata 17. Ethics of local audit approval were conducted as per national guidance.

## 3 Results

Case mix and access: 47 patients were treated—28 at the central and 19 at district hospitals (Table 2). Motorcycle‐related injuries comprised 42.6%. Central cases were more complex (Gustilo IIIA/B 69.6% vs. 15.8%). Time from injury to surgery was shorter at the central site (median 1 day) than districts (2 days). Preoperative antibiotics were administered in 100% versus. 47% of cases, respectively.

**TABLE 2:** Demographics and injury characteristics by the hospital type.

Characteristic	Central (*n* = 28)	District (*n* = 19)	Total (*N* = 47)
Male sex—*n* (%)	25 (89%)	17 (89%)	42 (89%)
Age—mean (years)	32.3	32.3	32.3
Motorcycle‐related injury—*n* (%)	12 (43%)	8 (42%)	20 (42.6%)
Gustilo IIIA/B—*n* (%)	16 (69.6%)	3 (15.8%)	19 (40.4%)
Time injury→surgery—median (days)	1	2	—
Pre‐op antibiotics—*n* (%)	28 (100%)	9 (47%)	37 (78.7%)
Open tibia shaft fracture—*n* (%)	28 (100%)	19 (100%)	47 (100%)

**FIGURE 1:**
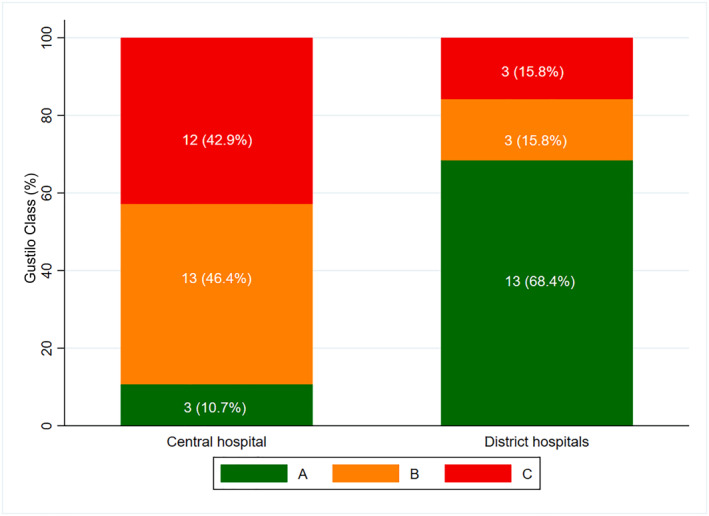
Gustilo Anderson open fracture classification grade among injury cases stratified by the treating hospital (*N* = 47). Stacked bar chart showing the distribution of AO/OTA fracture classifications (A, B, and C) at the central hospital versus district hospitals. At the central hospital (*n* = 28)—Type A 3 (10.7%), Type B 13 (46.4%), and Type C 12 (42.9%). At district hospitals (*n* = 19)—Type A 13 (68.4%), Type B 3 (15.8%), and Type C 3 (15.8%).

**FIGURE 2:**
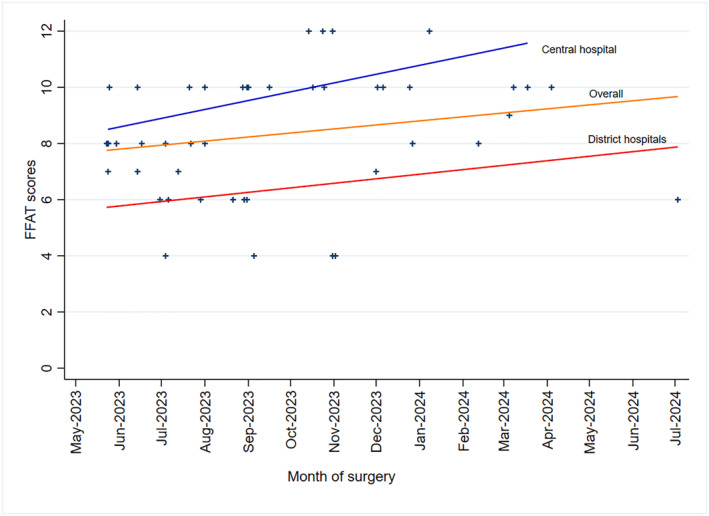
Distribution of FFATEF scores by the type of health facility and the trend over time. Scatter plot with linear trend lines showing FFATEF scores (*y*‐axis, 0–12) over time from May 2023 to July 2024 (*x*‐axis). Central hospital scores (blue) show an upward trend (*ρ* = 0.52 and *p* = 0.005); overall scores (orange) show moderate improvement, whereas district hospital scores (red) show no significant temporal change (*ρ* = 0.15 and *p* = 0.540).

Technical quality: Median FFATEF scores were higher at the central hospital (10.0 [IQR 9–11]) than district hospitals (6.0 [7, 8, 10, 11] and *p* < 0.001; Figure 1, and Table 3). Satisfactory scores (≥ 8) occurred in 26/28 (93%) central versus 6/19 (32%) district cases. Across domains, central cases scored higher for reduction, stability, implantation, and surgical impression (all *p* < 0.01). Central performance improved over time (*ρ* = 0.52 and *p* = 0.005); district scores showed no temporal change (*ρ* = 0.15 and *p* = 0.540; Figure 2).

**TABLE 3:** Technical quality outcomes (FFATEF) by the hospital type.

Outcome	Central	District
Total FFATEF—median (IQR)	10 (9–11)	6 (5–8)
Satisfactory (≥ 8)—*n* (%)	26/28 (93%)	6/19 (32%)
Trend over time (Spearman *ρ*, *p*)	0.52; 0.005	0.15; 0.540

Regression: Treatment at the central hospital was independently associated with higher odds of satisfactory FFATEF (OR 28.5, 95% CI 5.2–156.3, and *p* < 0.001), whereas higher Gustilo grade reduced the odds (OR 0.42 per grade, 95% CI 0.19–0.93, and *p* = 0.032; Table 4).

**TABLE 4:** Factors associated with satisfactory technical performance (FFATEF ≥ 8).

Predictor	Odds ratio	95% CI	*p*‐value
Central hospital (vs. district)	28.5	5.2–156.3	< 0.001
Gustilo grade (per grade increase)	0.42	0.19–0.93	0.032

*Note:* FFATEF domains scored 0–3 and total 0–12 with satisfactory defined a priori as ≥ 8. Unless otherwise indicated, values are *n* (%). Continuous variables are presented as mean (SD). Values are medians (IQR) or *n* (%), as appropriate. Odds ratios (OR) with 95% confidence intervals from logistic regression.

## 4 Discussion

This evaluation suggests that task sharing for open tibia external fixation can achieve satisfactory technical quality when delivered within integrated teams and adequate infrastructure, consistent with recent efforts to improve open fracture care in Malawi [20], but district‐level performance remained suboptimal despite training and remote reviews. Contributors likely include case volume and exposure, equipment availability (e.g., lack of fluoroscopy), team support, and inconsistent referral adherence. These findings support a shift from simple task shifting to true task sharing resulting in progressive scope, stronger mentoring cadence, and institutional prerequisites.

Policy and program implications involve (1) graduated implementation focusing initially on lower complexity cases, (2) minimum infrastructure standards (lighting, sterilization, instrumentation, and imaging where feasible), (3) defined mentoring intensity (e.g., weekly case reviews and periodic on‐site supervision), (4) real‐time quality monitoring using concise tools adaptable for local use and (5) robust referral networks matched to facility capability. Economic assessment is needed to compare decentralized care plus support versus strengthened centralization and transport systems.

Limitations include modest sample size, nonrandomized design, process‐oriented primary outcome, and lack of infection/union/function endpoints; future studies should assess long‐term functional outcomes as demonstrated elsewhere in Malawi [21]. FFATEF requires validation against patient outcomes and streamlining for routine adoption.

## 5 Conclusions

Task sharing for complex orthopedic trauma requires more than short‐course training. Central hospital embedding with integrated teams was associated with high rates of satisfactory technical performance, whereas district hospitals lagged in technical performance. Safe scale‐up will likely depend on institutional prerequisites, intensified mentoring, adherence to referral criteria, and continuous quality assurance. Future work should validate FFATEF against clinical outcomes and evaluate cost‐effectiveness of delivery models.

## Author Contributions

L.B. and O.B.: investigation, supervision, writing – review and editing. W.H.: conceptualization, methodology, supervision, writing – review and editing. F.M.: formal analysis, validation, writing – review and editing. C.M.: funding acquisition, project administration, writing – original draft and review.

## Funding

Johnson & Johnson Foundation Long Bone Fracture Program, KidsOR UK, and AO Alliance Foundation; funders had no role in study design, data collection, analysis, or reporting.

## Ethics Statement

Local audit approval from Kamuzu Central Hospital Research Committee (KCHRC/001/23) was conducted in accordance with the Declaration of Helsinki, Good Clinical Practices, and national guidance.

## Consent

The authors have nothing to report.

## Conflicts of Interest

The authors declare no conflicts of interest.

## Data Availability

Deidentified data are available from the corresponding author on reasonable request and with appropriate approvals.

## References

[1] J. G. Meara , A. J. Leather , L. Hagander , et al., “Global Surgery 2030: Evidence and Solutions for Achieving Health, Welfare, and Economic Development,” Lancet 386, no. 9993 (2015): 569–624, 10.1016/s0140-6736(15)60160-x. 25924834

[2] P. Joosten , M. Noorlander‐Borgdorff , M. Botman , M. B. Bouman , D. Embden , and G. Giannakopoulos , “Comparing Outcomes Following Direct Admission and Early Transfer to Specialized Trauma Centres in Open Tibial Fracture Treatment: A Systematic Review and meta‐analysis,” European Journal of Trauma and Emergency Surgery 50 (2023): 1–10, 10.1007/s00068-023-02366-x. PMC11035412 37776341

[3] N. P. Raykar , R. R. Yorlets , C. Liu , et al., “A Qualitative Study Exploring Contextual Challenges to Surgical Care Provision in 21 LMICs,” supplement, Lancet 385, no. S2 (2015): S15, 10.1016/s0140-6736(15)60810-8. PMC4803468 26313061

[4] K. Mwafulirwa , R. Munthali , I. Ghosten , and A. Schade , “Epidemiology of Open Tibia Fractures Presenting to a Tertiary Referral Centre in Southern Malawi: A Retrospective Study,” Malawi Medical Journal 34, no. 2 (2022): 118–122, 10.4314/mmj.v34i2.7. 35991814 PMC9356523

[5] W. Alemayehu , M. Melese , A. Bejiga , et al., “Surgery for Trichiasis by Ophthalmologists Versus Integrated Eye Care Workers: A Randomized Trial,” Ophthalmology 111, no. 3 (2004): 578–584, 10.1016/j.ophtha.2003.06.030. 15019339

[6] J. Gajewski , M. Cheelo , L. Bijlmakers , J. Kachimba , C. Pittalis , and R. Brugha , “The Contribution of Non‐Physician Clinicians to the Provision of Surgery in Rural Zambia—A Randomized Controlled Trial,” Human Resources for Health 17, no. 1 (2019): 60, 10.1186/s12960-019-0398-9. 31331348 PMC6647149

[7] C. McCord , G. Mbaruku , C. Pereira , C. Nzabuhakwa , and S. Bergstrom , “The Quality of Emergency Obstetrical Surgery by Assistant Medical Officers in Tanzanian District Hospitals,” Health Affairs 28, no. 5 (2009): w876–w885, 10.1377/hlthaff.28.5.w876. 19661113

[8] T. J. Wilhelm , I. K. Thawe , B. Mwatibu , H. Mothes , and S. Post , “Efficacy of Major General Surgery Performed by Non‐physician Clinicians at a Central Hospital in Malawi,” Tropical Doctor 41, no. 2 (2011): 71–75, 10.1258/td.2010.100272. 21303987

[9] T. J. Wilhelm , H. Mothes , D. Chiwewe , B. Mwatibu , and G. Kähler , “Delegating Endoscopy to Non‐Physician Clinicians in Malawi: Feasibility and Safety,” Endoscopy 44, no. 2 (2012): 174–176, 10.1055/s-0031-1291446. 22068703

[10] N. Mkandawire , C. Ngulube , and C. Lavy , “Orthopaedic Clinical Officer Program in Malawi: A Model for Providing Orthopaedic Care,” Clinical Orthopaedics and Related Research 466, no. 10 (2008): 2385–2391, 10.1007/s11999-008-0366-5. 18633684 PMC2584281

[11] T. J. Wilhelm , K. Dzimbiri , V. Sembereka , M. Gumeni , O. Bach , and H. Mothes , “Task‐Shifting of Orthopaedic Surgery to Non‐Physician Clinicians in Malawi: Effective and Safe?,” Tropical Doctor 47, no. 4 (2017): 294–299, 10.1177/0049475517717178. 28682219

[12] Johnson & Johnson Foundation , Bringing Orthopaedic Surgical Care Closer to Home for Malawi's Rural Population (Johnson & Johnson, 2024): [Internet], https://www.jnj.com/global‐health‐equity/bringing‐orthopedic‐surgical‐care‐closer‐to‐home‐for‐malawis‐rural‐population.

[13] AO Alliance Foundation , Training on External Fixation for Orthopaedic Clinical Officers in Malawi (AO Alliance, 2023): [Internet], https://ao‐alliance.org/training‐on‐external‐fixation‐for‐orthopedic‐clinical‐officers‐in‐malawi‐3/.

[14] A. T. Schade , M. Yesaya , J. Bates , C. Martin Jr , and W. J. Harrison , “The Malawi Orthopaedic Association/AO Alliance Guidelines and Standards for Open Fracture Management in Malawi: A National Consensus Statement,” Malawi Medical Journal 32, no. 3 (2020): 112–118, 10.4314/mmj.v32i3.2. 33488981 PMC7812144

[15] M. Sabawo , Z. Jaffry , L. Chokotho , and A. T. Schade , “An Assessment of Open Fracture Management in Hospitals in Malawi Before and Immediately After Implementing Open Fracture Guidelines,” JBJS Open Access 9, no. 2 (2024): e23: 00078, 10.2106/jbjs.oa.23.00078. PMC10984658 38572496

[16] D. H. Hawkes and W. J. Harrison , “Critiquing Operative Fracture Fixation: The Development of an Assessment Tool,” European Journal of Orthopaedic Surgery and Traumatology 27, no. 8 (2017): 1083–1088, 10.1007/s00590-017-1943-7. 28331965

[17] D. Hillier , D. Hawkes , P. Kenyon , and W. J. Harrison , “Fracture Fixation Assessment Tool Score (FFATS): How Does the Score Correlate With Surgeon Training Grade?,” supplement, Orthopaedic Proceedings 99‐B, no. S14 (2017): 1, 10.1302/1358-992X.99BSUPP_14.WOC-UK2017-001.

[18] E. G. Meinberg , J. Agel , C. S. Roberts , M. D. Karam , and J. F. Kellam , “Fracture and Dislocation Classification Compendium‐2018,” Journal of Orthopaedic Trauma 32, no. Suppl 1 (2018): S1–S170, 10.1097/BOT.0000000000001063. 29256945

[19] R. B. Gustilo and J. T. Anderson , “Prevention of Infection in the Treatment of One Thousand and twenty‐five Open Fractures of Long Bones,” Journal of Bone & Joint Surgery 58, no. 4 (1976): 453–458, 10.2106/00004623-197658040-00004. 773941

[20] A. T. Schade , M. Sabawo , Z. Jaffry , et al., “Improving the Management of Open Tibia Fractures, Malawi,” Bulletin of the World Health Organization 102, no. 4 (2024): 255–264, 10.2471/blt.23.290755. 38562195 PMC10976873

[21] A. T. Schade , M. Yesaya , K. Mwafulirwa , et al., “Functional Outcomes and Quality of Life at 1‐Year Follow‐Up After an Open Tibia Fracture in Malawi: A Multicentre, Prospective Cohort Study,” Lancet Global Health 11, no. 10 (2023): e1609–e1618, 10.1016/S2214-109X(23)00346-7. 37666261 PMC10509037

